# Exploring the uncertainties of early detection results: model-based interpretation of mayo lung project

**DOI:** 10.1186/1471-2407-11-92

**Published:** 2011-03-07

**Authors:** Lu Shi, Haijun Tian, William J McCarthy, Barbara Berman, Shinyi Wu, Rob Boer

**Affiliations:** 1 Department of Health Services, 650 Charles E. Young Drive S. 61-253 CHS, Los Angeles, CA 90095, USA; 2 Health Benchmarks, Inc, IMS Health, 21650 Oxnard St, Ste 550, Woodland Hills, CA, USA; 3 UCLA Division of Cancer Prevention and Control Research, Los Angeles, CA, USA; 4 Epstein Department of Industrial and Systems Engineering, University of Southern California, Los Angeles, CA, USA; 5 Erasmus MC, University Medical Center Rotterdam, The Netherlands

## Abstract

**Background:**

The Mayo Lung Project (MLP), a randomized controlled clinical trial of lung cancer screening conducted between 1971 and 1986 among male smokers aged 45 or above, demonstrated an increase in lung cancer survival since the time of diagnosis, but no reduction in lung cancer mortality. Whether this result necessarily indicates a lack of mortality benefit for screening remains controversial. A number of hypotheses have been proposed to explain the observed outcome, including over-diagnosis, screening sensitivity, and population heterogeneity (initial difference in lung cancer risks between the two trial arms). This study is intended to provide model-based testing for some of these important arguments.

**Method:**

Using a micro-simulation model, the MISCAN-lung model, we explore the possible influence of screening sensitivity, systematic error, over-diagnosis and population heterogeneity.

**Results:**

Calibrating screening sensitivity, systematic error, or over-diagnosis does not noticeably improve the fit of the model, whereas calibrating population heterogeneity helps the model predict lung cancer incidence better.

**Conclusions:**

Our conclusion is that the hypothesized imperfection in screening sensitivity, systematic error, and over-diagnosis do not in themselves explain the observed trial results. Model fit improvement achieved by accounting for population heterogeneity suggests a higher risk of cancer incidence in the intervention group as compared with the control group.

## Background

Lung cancer is the leading cause of cancer deaths among men and women in the United States [1]. Clinical treatment has not led to major improvements in patients' survival from lung cancer. Although steps are being taken to prevent lung cancer incidence, particularly through anti-tobacco public health efforts, considerable time is required to realize a noticeable decrease in the population incidence of this disease. As a result, considerable attention has focused on the utility of screening as a strategy for achieving a reduction in lung cancer mortality.

The Mayo Lung Project (MLP), a randomized controlled trial including 9211 male cigarette smokers, was conducted between 1971 and 1983 to assess whether frequent screening through chest x-rays and sputum cytology administered every four months for a six-year period would result in a long-term reduction in lung cancer mortality. Control group participants received usual care at the Mayo Clinic, i.e., advice to receive these screening tests annually. No significant between-group difference was found in lung cancer mortality, at the end of the trial as well as at the end of an extended 20 year follow-up [2], when lung cancer mortality rates were defined as the number of lung cancer deaths divided by person-years at risk of death. However, there was a larger incidence of lung cancer in the screening intervention group, and those diagnosed with lung cancer in the screening intervention group did have longer lung cancer survival from the time of diagnosis than those diagnosed with lung cancer in the control group [2,3].

Considerable interest has focused on examining possible explanations for this survival benefit and lack of mortality benefit. It has been argued that the observed survival improvement could be due to some well-known mechanisms, i. e., over-diagnosis [4], lead-time bias, and length bias [5]. Other explanations of the MLP results include: the excess radiation risk received during the trial could offset the mortality benefit of the screening group [6]; a survival benefit did exist but was concealed by higher lung cancer risks in the screening intervention group [7]; there was not enough statistical power to demonstrate a mortality benefit due to design of the trial [3,8], etc. Below we discuss some of those key arguments from a modeling perspective.

### Over-diagnosis

Over-diagnosis is the detection of lesions that meet the pathologic definition of cancer but will never progress to cause symptoms or death during a patient's lifetime. An over-diagnosed cancer, then, is a tumor that can be screen-detected at preclinical stage II-, but never grows into III+. Thus, this tumor can never be clinically diagnosed until the patient dies from causes other than lung cancer. In other words, an over-diagnosed cancer is a tumor that could be detected by screening but will never cause clinical symptoms. Marcus et al [5] investigated this issue of over-diagnosis in MLP and concluded that better survival for individuals in the intervention arm indicates that some lesions with limited clinical relevance may have been identified in this group. In other words, the 46 excess cancers identified at the end of the study were in fact not morbid lesions or were so indolent that patients with significant health risks (e.g., smokers) were more likely to die of other causes before these cancers became a problem.

### Screening sensitivity and systematic negative error

Technological limits of chest X-rays might also have influenced the trial outcome. If a screening takes place when lung cancer is at a preclinical stage, there is a probability of detecting the cancer by the screening test ("test sensitivity"), depending on stage and cell type. In terms of test sensitivity, chest X-ray does not compare favorably with more recent screening tools such as spiral computed tomography (CT). A related concept is the systematic negative error, i. e., the probability of a second false-negative diagnosis given that the previous diagnosis is false-negative. Systematic errors from screening tests can occur as a result of person-level issues, lesion-level issues and timing issues.

### Initial difference in cancer risk

Strauss [7] found from the MLP data that exposure to air pollution was significantly associated with higher lung cancer incidence and mortality in the control arm whereas the same exposure predicted lower incidence and mortality in the screening intervention arm. In addition, history of bronchitis/pneumonia predicted lung cancer incidence in the screening intervention arm. The fact that these associations varied across the trial arms indicates that there might have been hidden differences between the two arms, particularly those differences in confounding variables that the trial did not measure and failed to balance across the two experimental conditions. Hence, the hypothesis that the initial lung cancer risk could be higher in the screening intervention arm than in the control arm. This hypothesis is also consistent with the fact that the screening intervention arm had more lung cancer incidence and lung cancer mortality while no biomedical evidence of over-diagnosis of lung cancer has been confirmed in this trial.

Newer technologies such as spiral computed tomography (CT) have been shown to identify early lung cancer lesions [9] and may prove to be a more useful screening modality than chest x-rays. However, if lung cancer lesions with little or no clinical relevance exist, CT will likely identify them at an even higher rate than would have occurred through use of the traditional chest x-ray. The sensitivity of newer technologies can have considerable clinical implications, underscoring the importance of gaining a better understanding of the plausibility of hypotheses such as sensitivities, over-diagnosis and initial difference in data from large scale trials such as the MLP. In this paper, we explore the relevance of over-diagnosis and initial difference hypotheses with a microsimulation approach, as well as the relevance of two screening parameters: test sensitivity and systematic negative error.

## Methods

MISCAN is a microsimulation model for the early detection of chronic diseases, where a large number of individual life histories are stochastically generated to simulate chronic disease progressions [10]. The MISCAN-lung cancer model has been developed to maintain a comprehensive surveillance of population trends in lung cancer and to estimate the impact of cancer control interventions [11]. In MISCAN-lung, the disease progression includes the following phases: initiation of cells, clonal expansion of initiated cells, malignant transformation, and progression to diagnosis. Preclinical lung cancer can pass through a number of disease stages, each with a specific dwelling time distribution, before a clinical diagnosis takes place. Stage distribution depends on transition probabilities to diagnosis versus progression to next disease state. In pre-invasive stages we use continuous growth by cell clone. When it turns into invasive cancer, we start to model the growth by discrete stages.

We calibrate the cancer incidence to the observed results from the Mayo Lung Project, while the onset of pre-clinical screen-detected cancer follows a Weibull distribution with its shape and mean parameters based upon the age and cell type-specific cancer incidence in the Surveillance, Epidemiology, and End Results (SEER) program. For the impact of screening, we specify an exponentially distributed sojourn time for the preclinical disease state (with the mean parameter calibrated to trial results), as earlier studies [12,13] have shown that the exponential distribution for the preclinical state provides a good fit to cancer incidence and prevalence. We simulate three separate pathways to clinical diagnosis for squamous, adeno/large cell, and small cell lung cancers. Each pathway starts with undetectable preclinical Stage I and can develop into screen-detectable Stage II. Or, the tumor can be clinically diagnosed in Stage III. The cancers that develop further are diagnosed as Stage IV, when the tumor will have metastasized. In other words, we assume that preclinical lung cancer can only be screen-detected while clinical lung cancer can be both screen-detected and clinically detected.

Our model specifies the screening policy and attendance pattern, as well as characteristics of screening tests, and for this study the subjects in the intervention-arm are simulated to receive screenings every four months for six years. The sensitivity of a screening test depends on the preclinical disease state (categorized by stage and cell type). If a lung cancer is detected by screening during the preclinical phases, the time of diagnosis is earlier than in the situation without screening. Thus the stage at diagnosis could be Stage I or II (to be referred to as "stage II-" hereinafter) at screen-detection, instead of Stage III or IV (to be referred to as "stage III+" hereinafter) at clinical diagnosis.

**Figure 1:**
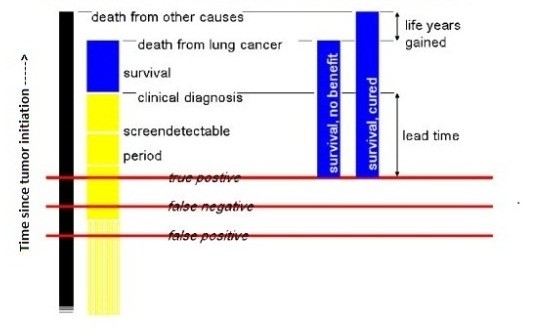
**A Diagram for the Model Flow of MISCAN-Lung**.

A diagram for the model flow is given in Figure 1 and a list of parameters used in MISCAN-lung is provided in Additional File 1.

We explore over-diagnosis and initial risk difference as possible explanations for the observations from the MLP, as well as the potential effectiveness of using more sensitive screening technologies. First, we model the impact of Stage II screening sensitivity and Stage III screening sensitivity, i.e., the probability of the positive result conditional on being in preclinical screen-detectable Stage II and the probability of the positive result conditional on being in screen-detectable Stage III. Next, we add a related parameter, systematic negative error. That is, if a cancer is missed through screening during the sojourn time there is an increase in the probability that the same cancer will be missed again at a next screening during the sojourn time. We term this incremental probability as systematic negative error.

We first set sensitivity parameters to be 1 and systematic negative error parameters to be 0 (Simple Model in Additional File 2), and obtain a deviance measure by subtracting the log likelihood of the estimated model from the log likelihood of the saturated model and multiplying the result by 2. We apply a χ2 test to the resulting deviance measure as a test of goodness of fit. We then fit for sensitivity parameters and set systematic negative error to zero (Sensitivity Model in Additional File 2) to see if this model outputs a deviance value significantly lower than the deviance measure from the Simple Model. Next, we set sensitivity parameters to be 1 and fit for the systematic negative error parameters (Systematic Error Model in Additional File 2) to test their effect on reducing the deviance. We then fit for both sensitivity parameters and systematic negative error parameters in order to test their joint effect (Sensitivity-Error Model in Additional File 2). For over-diagnosis in our study, we introduce a parameter for indolent cancers. If fitting this indolent cancer parameter does not decrease the deviance measure from our Simple Model, then the simulation result is inconsistent with an assumption of serious over-diagnosis (Over-diagnosis Model).

Finally, we test the hypothesis of differential lung cancer risks between the intervention group and the control group (Risk Difference Model) by calibrating a difference factor of lung cancer onset probability between the two trial arms. All previous models assumed that the lung cancer onset probability was the same between two trial arms, while this Risk Difference Model fits for a difference factor for the lung cancer onset probability. In other words, the Risk Difference Model allows the two trial arms to differ in their initial lung cancer risk. For the risk difference hypothesis, we also examined the participant's behavior to identify smoking status at the beginning of the intervention, running an independent t-test to compare the number of cigarettes smoked per day between the screening intervention arm and the control arm.

## Results

Additional File 2 lists the parameters fitted in our six different models. Predicted results from the Simple Model and observed results are summarized in Additional File 3, which shows that the Simple Model achieved a deviance measure of 23.45. While 90 screen-detected lung cancers and 116 otherwise diagnosed lung cancers were observed in the screening intervention arm, the Simple Model expects 88.1 screen-detected lung cancers and 99.7 otherwise diagnosed ones. A binomial test shows that the observed number of lung cancers in the screening intervention arm was significantly higher (p = 0.016) than what was expected by the Simple Model.

Additional File 4 summarizes the deviance reduction, a measure of how much the model has improved from the Simple Model to all of the other five models. The Sensitivity Model, where we fitted for sensitivity parameters at stage II- and stage III+, optimizes the sensitivity values to be 0.967 for stage II- and 0.995 for stage III+. The Sensitivity Model however did not substantially improve the model prediction from model 1, with a reduction of the deviance measure by 1.27 (Δdf = 1, p = 0.23).

In the Systematic Error Model we obtained fitted values of 0.022 and 0.185. This model moderately decreases the deviance from 23.54 in the Simple Model to 21.4, which implies that the systematic error parameter did not significantly affect the observed results (ΔDev = 2.14, Δdf = 1, p = 0.14). The Sensitivity-Error Model further added screening sensitivities as fitting parameters to the Systematic Error Model but did not improve the model prediction significantly (the deviance measure is 21.4 in Systematic Error Model vs. 21.38 in Sensitivity-Error Model). The comparison between the Simple Model and the Sensitivity Model, as well as the comparison between the Systematic Error Model and the Sensitivity-Error Model, indicate that the sensitivity parameters did not contribute significantly to the model improvement.

Our Over-diagnosis Model included the indolent cancer parameters in the model and found minimal deviance reduction (ΔDev = 0.07, Δdf = 1, p = 0.79). Our Risk Difference Model allows for the initial risk difference between the intervention groups and control groups and reduced the deviance from 23.54 in the Simple Model to 18.5, and thereby achieved the smallest deviance measure among all six models (ΔDev = 5.04, Δdf = 1, p = 0.02). Finally, with respect to smoking behavior at baseline, we found a clinically small but statistically significant between-group difference in number of cigarettes smoked per day, with screening intervention group subjects on average smoking .53 more cigarettes per day than subjects in the control group (t = 2.09, p = 0.03).

## Discussion and Conclusion

New screening technologies could focus on features that could detect tumors of smaller sizes, or they could focus on features that increase screening sensitivity within the current range of screen-detectable tumor sizes. If fitting the sensitivity measures had increased the model's prediction of observed lung cancer incidence by cell type and stage for both groups, we would expect that emerging technologies with higher screening sensitivity measures could achieve improved mortality outcomes. However, the Sensitivity Model, Systematic Error Model, and Sensitivity-Error Model did not substantially improve the fit from what had been achieved by the Simple Model. The implication is that the hypothesized imperfections in screening sensitivity alone could not have explained the lack of mortality benefit in the Mayo Lung Project. Thus, in evaluating the priorities of developing new screening technologies for lung cancer, it might be advisable to focus more on the features that could potentially lower the detectable threshold rather than on features that increase screening sensitivity within the current range of screen-detectable tumor sizes.

The failure of our Over-diagnosis Model to significantly improve the fit from the Simple Model also leads us to conclude that over-diagnosis is not likely to be a serious issue in MLP. This confirms the observation that over-diagnosis bias is minimal in screen-detected lung cancers in the case of chest radiograph examination [14]. However, findings from studying MLP chest X-ray screening in the 1970 s do not necessarily imply that over-diagnosis is an irrelevant consideration for emerging technologies such as spiral computed tomography. Inasmuch as CT detects smaller tumors than do chest X-rays, there may well be more chances for the former to give false positive results and thus produce artificially "better" lung cancer survival through over-diagnosis. The plausibility of over-diagnosis of lung cancer diagnosis when using newer, more sensitive screening technologies can be tested using the MISCAN micro-simulation model we used here, so long as the modeler has access to a similar set of input parameters.

The significant improvement achieved by the Risk Difference Model suggests that there might have been an a priori higher risk of cancer incidence in the intervention than in the control group, i. e., the mortality benefit from screening was concealed by the higher lung cancer risk in the intervention group. This is consistent with the view that population heterogeneity could have played a role in the trial outcomes [15]. However, Marcus and Prorok [16] showed that adjustment for four lung cancer risk factors (age at entry, history of cigarette smoking, exposure to non-tobacco lung carcinogens, or previous pulmonary illnesses) did not alter the original findings of the Mayo Lung Project. Another explanation could be that there might have been a coincidental imbalance in the percentages of small cell lung cancer between the two trial arms, since small cell lung carcinoma progresses much faster than the majority of non-small cell lung carcinoma cases, and thus a different proportion of small-cell cases would be very likely to result in different mortality outcomes [17].

Although we find a statistically significant difference in the number of cigarettes smoked per day, there is no documented evidence that a difference of one half of a cigarette a day will make a significant difference in health outcomes. Rather, this difference might have reflected a "volunteer effect" [18] since in MLP only those randomized to the screening intervention group were asked about their consent to participate in the trial. Ideally, the trial's randomization process should have occurred after rather than before the baseline prevalence screening, since the prevalence screening could have created behavioral differences between the two trial arms.

Despite the significant improvement in model fit achieved by the Risk Difference Model, from the perspective of model parsimony our Simple Model might still be the best choice from among all six models discussed here, especially if we seek to extend MISCAN to simulations of other lung cancer screening programs that do not suffer from suboptimal randomization of subjects to experimental condition.

Finally, in considering the utility of interventions for preventing chronic diseases, the effectiveness of screening and testing programs should not be measured only in terms of the extent to which earlier detection can achieve earlier treatment. Rather, as Huuskonen [19] proposed, screening should be viewed as a coordinated intervention with the goal of identifying populations at risk and modifying that risk. Counseling and pharmacological interventions have been added to screening interventions in lung cancer detection programs [20,21]. Meanwhile, as reduced risk for this disease may begin as early as five years after smoking cessation, evaluation of the lung cancer screening program will be complete only when the trial subjects' smoking behavior is measured on at least an annual basis. Insufficient information about changes in smoking behavior among MLP trial subjects after the six-year intervention limits the investigation of long-term behavioral differences between the two trial arms. Current and future clinical trials of the impact of lung cancer screening on mortality should incorporate measurement of smoking behavior at regular follow-up intervals into their data collection process.

In this study, modeling was used to try to represent our knowledge of the system of interaction between the biological and screening processes. While we have, in principle, full knowledge of the screening processes because these are designed by people, we are less confident about our model's ability to represent the biological processes. A large part of the biological processes were not observed, in this case particularly because detection of a cancer triggered interventions that made it impossible to determine whether the cancer was indolent after all. Modeling is one way to learn some more about the underlying biological processes in a more indirect way.

This study investigated a number of hypotheses that have been raised in the literature. The true hypothesis may not be among them. Moreover, the hypotheses that have been raised in the literature are generally not specified exactly therefore we were left to make some arbitrary assumptions, e.g. to define what is exactly the behavior of an indolent cancer. Also, this study has only looked at the main effects of risk difference and over-diagnosis on incidence outcome, i.e., these parameters were mainly considered as single factors rather than factors that could interact with other parameters to influence the outcome. If large population datasets for cancer screening become available, it will be interesting for future studies to examine the possible interaction effects of sensitivity and risk difference, systematic error and risk difference, or a possible three-way interaction of sensitivity-systematic error-risk difference.

## Competing interests

The authors declare that they have no competing interests.

## Authors' contributions

LS carried out the modeling and drafted the manuscript. HT did the initial round of modeling and contributed part of the literature review. WJM participated in the literature review and helped with revisions. BB participated in the literature review and helped with revisions. SW helped with the modeling. RB conceived of the study and helped with the revisions. All authors read and approved the final manuscript.

## Pre-publication history

The pre-publication history for this paper can be accessed here:

http://www.biomedcentral.com/1471-2407/11/92/prepub

## Supplementary Material

Additional file 1 Appendix 1: MISCAN-lung Model Profile as applied in Mayo Lung Project. Click here for file

Additional file 2 Table 1: Fitting the Six Models on Chest X-ray Screening in the Mayo Lung Trial. Click here for file

Additional file 3 Table 2: Comparison of observed and modeled results of cancers detection in the MLP. Click here for file

Additional file 4 Table 3: Calibrated Parameters and Deviance from Observed Detections in Six Different MISCAN Models. Click here for file
