# Supplementary material for: Exploring the uncertainties of early detection results: model-based interpretation of mayo lung project
Source: BMC Cancer. 2011 Mar 7;11:92. doi: 10.1186/1471-2407-11-92 (PMC3058105; doi:10.1186/1471-2407-11-92)
Supplement: Additional file 1 — Appendix 1: MISCAN-lung Model Profile as applied in Mayo Lung Project. [file 1471-2407-11-92-S1.DOC]

**Appendix 1: MISCAN-lung Model Profile as applied in Mayo Lung Project**

Parameters for the onset of preclinical screen-detectable cancer (Weibull distribution)[1]

| Shape parameter | 7.7851 |
| --- | --- |
| Mean of Squamous cell | 73.1931 |
| Mean of adeno/large cell | 70.1529 |
| Mean of small cell | 60.6221 |

1] based on age and cell type specific incidence in SEER

Years of duration in pre-clincal screen-detectable cancer disease states (exponential distribution) (calibrated to trial results)

| Mean duration of non-small cell II- | 1.4695 |
| --- | --- |
| Mean duration of non-small cell III+ | 3.1819 |
| Mean duration of small cell II- | 0.3225 |
| Mean duration of small cell III+ | 0.9104 |

Systematic error of screening test conditional on being in a preclinical screen-detectable stage of lung cancer

| systematic error at Stage II | 0.3536 |
| --- | --- |
| systematic error at Stage III | 0.1194 |

Cell type ractions diagnosed in early stages

| Squamous Stage II- | 27.61% |
| --- | --- |
| Adeno/large Stage II- | 26.05% |
| Small cell Stage II- | 10.98% |

Lung cancer survival and other-cause mortality by cell-type and stage of diagnosis

| Cell type/Stage | Shape of Weibull survival | Mean of Weibull survival | Other-cause mortality |
| --- | --- | --- | --- |
| Squamous Stage II- | 0.573 | 2.419 | .18 |
| Squamous Stage III+ | 0.641 | 0.752 | .06 |
| Adeno/large Stage II- | 0.676 | 4.783 | .29 |
| Adeno/large Stage III+ | 0.607 | 0.674 | .05 |
| Small cell Stage II- | 0.727 | 1.049 | .08 |
| Small cell Stage III+ | 0.738 | 0.507 | .01 |
