# Supplementary material for: Exploring the uncertainties of early detection results: model-based interpretation of mayo lung project
Source: BMC Cancer. 2011 Mar 7;11:92. doi: 10.1186/1471-2407-11-92 (PMC3058105; doi:10.1186/1471-2407-11-92)
Supplement: Additional file 2 — Table 1: Fitting the Six Models on Chest X-ray Screening in the Mayo Lung Trial. [file 1471-2407-11-92-S2.DOC]

**Table 1:**

Fitting the Six Models on Chest X-ray Screening in the Mayo Lung Trial

| Models  Parameters fitted | Simple | Sensitivity | Systematic error | Sensitivity & Systematic error | Indolent cancer | Risk difference |
| --- | --- | --- | --- | --- | --- | --- |
| Systematic error [[1]](#footnote-2)  at Stage 2 | 0 | 0 | Fit | Fit | 0 | 0 |
| Systematic error  at Stage 3 | 0 | 0 | Fit | Fit | 0 | 0 |
| Sensitivity [[2]](#footnote-3)  at Stage 2 | 1 | Fit | 1 | Fit | 1 | 1 |
| Sensitivity  at Stage 3 | 1 | Fit | 1 | Fit | 1 | 1 |
| Indolent cancer[[3]](#footnote-4) | 0 | 0 | 0 | 0 | Fit | 0 |
| Risk difference at the baseline[[4]](#footnote-5) | 0 | 0 | 0 | 0 | 0 | Fit |

1. The probability that having a false-negative result elevates the risk of having false-negative results again at later screenings. [↑](#footnote-ref-2)
2. the probability of the positive result conditional on being in a certain stage of cancer progression [↑](#footnote-ref-3)
3. The probability that a tumor will never progress into advanced stages [↑](#footnote-ref-4)
4. The factor that the screening intervention arm has higher probability to develop lung cancer [↑](#footnote-ref-5)
