# Supplementary material for: Exploring the uncertainties of early detection results: model-based interpretation of mayo lung project
Source: BMC Cancer. 2011 Mar 7;11:92. doi: 10.1186/1471-2407-11-92 (PMC3058105; doi:10.1186/1471-2407-11-92)
Supplement: Additional file 3 — Table 2: Comparison of observed and modeled results of cancers detection in the MLP. [file 1471-2407-11-92-S3.DOC]

**Table 2:** Comparison of observed and modeled results of cancers detection in the MLP

|  | INTERVENTION ARM | CONTROL ARM |
| --- | --- | --- |
|  | Observed Predicted | Observed Predicted |

Mode of detection

| Second screening | 5 | 5.3 |  |  |
| --- | --- | --- | --- | --- |
| Later screening | 85 | 82.8 |  |  |
| **Interval cancers by year** |  |  |  |  |
|
| 1^st^ | 35 | 28.3 | 5 | 5.5 |
| 2^nd^ | 20 | 16.8 | 12 | 12.3 |
| 3^rd^ | 20 | 15.3 | 19 | 15.4 |
| 4^th^ | 12 | 12.3 | 12 | 17.4 |
| 5^th^ | 11 | 8.8 | 18 | 19.4 |
| 6^th^ and later | 18 | 18.2 | 94 | 113.6 |
|  |  |  |  |  |
| **Cancers by cell type & stage at routine screening** |  |  |  |  |
| Squamous II- | 30.9 | 24.4 |  |  |
| Squamous III+ | 7 | 7 |  |  |
| Adeno/large II- | 25.8 | 29.7 |  |  |
| Adeno/large III+ | 6 | 8.6 |  |  |
| Small II- | 10.3 | 10 |  |  |
| Small III+ | 10 | 10.3 |  |  |
| **Otherwise diagnosed cancers** |  |  |  |  |
| Detected during the six years of intervention | 16 | 18.7 | 23 | 23.3 |
| Detected during follow-up years | 27 | 26.6 | 25 | 24.4 |
|  |  |  |  |  |
