# Supplementary material for: Exploring the uncertainties of early detection results: model-based interpretation of mayo lung project
Source: BMC Cancer. 2011 Mar 7;11:92. doi: 10.1186/1471-2407-11-92 (PMC3058105; doi:10.1186/1471-2407-11-92)
Supplement: Additional file 4 — Table 3: Calibrated Parameters and Deviance from Observed Detections in Six Different MISCAN Models. [file 1471-2407-11-92-S4.DOC]

**Table 3:**

**Calibrated Parameters and Deviance from Observed Detections in Six Different MISCAN Models**

| Models  Parameters fitted | Simple | Sensitivity | Systematic error | Sensitivity-error | Indolent cancer | Risk difference |
| --- | --- | --- | --- | --- | --- | --- |
| Systematic error  (Stage 2) |  |  | 0.022 | 0.022 |  |  |
| Systematic error  (Stage 3) |  |  | 0.185 | 0.189 |  |  |
| Sensitivity  (Stage 2) |  | 0.967 |  | 0.938 |  |  |
| Sensitivity  (Stage 3) |  | 0.995 |  | 1 |  |  |
| Indolent cancer |  |  |  |  | 0.001 |  |
| Risk difference |  |  |  |  |  | 0.232 |
| Deviance from  observed detection | 23.54 | 22.27 | 21.4 | 21.38 | 23.47 | 18.5 |
| Deviance reduction from Simple |  | 1.27 | 2.14 | 2.16 | 0.07 | **5.04**** |
